# Continuous Carbonation for Synthesis of Pseudo-Boehmite by Using Cross-Flow Rotating Packed Bed through the Reaction of NaAlO_2_ Solution with CO_2_ Gas

**DOI:** 10.3390/nano10020263

**Published:** 2020-02-04

**Authors:** Xiaojing Ren, Youzhi Liu, Longxin Miao

**Affiliations:** Shanxi Province Key Laboratory of Higee-Oriented Chemical Engineering, North University of China, No.3, Xueyuan Road, Jiancaoping District, Taiyuan 030051, China; rxj123321@126.com (X.R.);

**Keywords:** pseudo-boehmite, cross-flow rotating packed bed, continuous carbonation, final pH

## Abstract

This research established a novel method for the preparation of pseudo-boehmite (PB) via a continuous carbonation of CO_2_ gas and a NaAlO_2_ solution in a cross-flow rotating packed bed (CF-RPB). In the CF-RPB, the NaAlO_2_ solution can be sheared into fine liquid filaments and droplets, and react in full contact with the CO_2_ gas. Effects of synthesis parameters, including the concentration of the NaAlO_2_ solution, the gas–liquid ratio, the rotating speed of the CF-RPB, and the final pH of the solution on the crystal structure of PB, were fully investigated. A series of characterizations, including X-ray diffraction (XRD), scanning electron microscopy (SEM), transmission electron microscopy (TEM) and Brunauer–Emmett–Teller (BET) analysis, were carried out to explain the evaluation results and to find the relationship between PB properties and the synthetic conditions. The results showed that PB with a high specific surface area (495 m^2^/g) and large pore volume (2.125 cc/g) can be obtained when the concentration of the NaAlO_2_ solution was 0.1 mol/L, the gas–liquid ratio was 3:1, the rotating speed of RPB was 600 rpm, and the final pH was around 10.5. PB obtained by this method had a higher quality compared with that using a stirred tank reactor. Moreover, the continuous carbonation can be efficiently batch-produced, which provided a new idea for an industrial application.

## 1. Introduction

Pseudo-boehmite (PB) is a kind of aluminum hydroxides with poor crystallization, which possesses high mechanical strength, a large specific surface area, and abundant pore volume. It could be the precursor of *γ*-Al_2_O_3_ and the binder for a Fluid Catalytic Cracking (FCC) catalyst [1,2]. With the increasing proportion of residue in FCC (Fluid Catalytic Cracking) feed stock, traditional PB, due to a low specific surface area and narrow pore size distribution, cannot meet the request for actual production. Therefore, the low-cost preparation method for PB with a large pore volume and high specific surface area needs to be developed [3].

The preparation methods of PB are mainly sol–gel [4,5,6], hydrothermal method [7], alcoholic aluminum method [8], coprecipitation method [9], and carbonation method [10]. According to a previous study, the carbonation of a NaAlO_2_ solution and CO_2_ gas has become one of the most economical technologies to produce PB owing to its green manufacturing process and low cost. Moreover, the raw materials are by-products from alumina technology [11].

The reaction principles for carbonation can be described by the following equations [10]:

(1) $$2NaOH(aq)+CO_{2}(g)\to Na_{2}CO_{3}(aq)+H_{2}O(aq),$$

(2) $$2NaAlO_{2}(aq)+CO_{2}(g)+H_{2}O(aq)\to 2Al{\left(OH\right)}_{3}(s)+Na_{2}CO_{3}(aq),$$

(3) $$NaAlO_{2}(aq)+2H_{2}O(aq)\to Al{\left(OH\right)}_{3}(s)+NaOH(aq),$$

(4) $$Al{\left(OH\right)}_{3}(s)+CO_{2}(g)+Na_{2}CO_{3}(aq)\to 2NaAl{\left(OH\right)}_{2}CO_{3}(s)+H_{2}O(aq).$$

With the consumption of CO_2_ gas, the pH value of the aqueous phase gradually decreases to 10.5, the reaction stops with the target product (PB) obtained in the reaction (2) and the impurity Bayerite obtained in reaction (3). In order to improve the reaction efficiency (2), it is particularly important to reduce gas–liquid mass transfer resistance and intensify the reaction between CO_2_ and the aqueous phase. A traditional carbonation process occurs in a stirred tank reactor by bubbling CO_2_ into a NaAlO_2_ solution in which CO_2_ gas cannot be used completely and distributed evenly in the aqueous phase, thereby resulting in a long reaction time, wide pore size distribution, and more impurities [12].

Therefore, some researchers have made efforts to produce PB by reducing gas–liquid mass transfer. For example, Wang et al. [12] prepared PB with a large pore volume (2.22 cc/g) and a high specific surface area (548.5 m^2^/g) by a membrane-dispersion microstructured reactor. To improve the transfer efficiency, the CO_2_ gas dispersed into many microporous bubbles and reacted with liquid at the surface of the membrane. However, this method has a low processing capacity causing difficulties when exerted into industrial production. High-gravity technology [13,14,15] is one of the most promising branches of process intensification (PI), which is carried out in a rotating packed bed (RPB) and has been applied to deaeration, distillation, reaction, absorption, mass transfer, nanoparticles preparation, and so on. The high-speed rotating packing driven by the motor, shears the liquid into tiny droplets, filaments, and liquid membranes, as well as the gas into gas bubbles, which provides intense micromixing and strengthens the mass transfer process. In this way, the particle size distribution (PSD) of the product can be controlled. As a result, the gas–liquid mass transfer is increased by 10–100 times and the size of the equipment is reduced, thereby decreasing the operating costs. High-gravity technology has been successfully applied to the preparation of nanomaterials [16], such as nano-calcium carbonate [17], nano-barium sulfate [18], nano-zinc oxide [19], and so on. Guo et al. [20,21] prepared fibrous PB with a diameter of 1–10 nm and a length of 100–300 nm by high-gravity technology. According to previous research, it is found that the carbonation time in the high-gravity environment is nearly by half shorter than that in the traditional stirred tank reactor, and the processing capacity is improved six times [22]. A series of studies has shown that low CO_2_ utilization and a long carbonation time can be solved by high-gravity technology. However, there are still some problems in the reaction process that need to be tackled. For example, an inconsistent nucleation time caused by the mixing of raw materials with PB will result in a wide particle size distribution and low purity of the product. Furthermore, the reproducibility in mass production will also be reduced.

**Figure 1:**
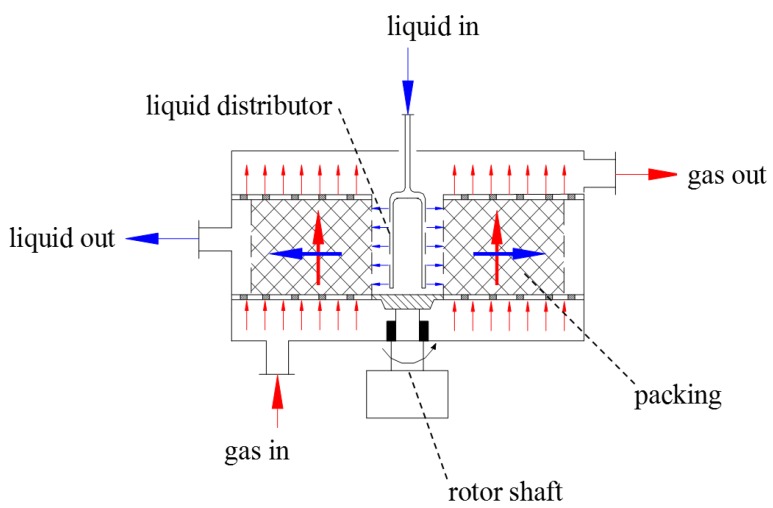
Main structure of a cross-flow rotating packed bed (RPB).

In order to solve the above problems, the continuous carbonation for PB synthesis by high-gravity technology was proposed for the first time in this paper. Cross-flow PPB (CF-RPB) [23,24] is a type of RPB in which gas and liquid react in a cross-flow contact in PRB. Due to this type of contact, the CF-RPB could be operated at a higher gas or liquid flow rate because of the low tendency of flooding compared with traditional RPB, which would be more applicable for industrialization. The main structure of CF-RPB is shown in Figure 1. The continuous preparation of PB with a high specific surface area and large volume by high-gravity continuous carbonation was completed through non-circulating gas–liquid continuous feeding, using CF-RPB as a carrier. The effects of the concentration of the NaAlO_2_ solution, gas–liquid ratio, and rotating speed of CF-RPB on PB pore characteristics were systematically studied.

## 2. Materials and Methods

### 2.1. Materials

Carbon dioxide (CO_2_) gases were supplied with a purity of over 99.995%. Sodium aluminate (NaAlO_2_, 99.9% in purity) was received from the Guangfu Fine Chemical Research Institute.

### 2.2. Experimental Devices and Process

The main parameters of CF-RPB are listed in Table 1. The liquid enters the packed bed from a liquid distributor and sprays onto the inner edge of the backed bed. The liquid moves outward through the packing due to centrifugal force, splashes onto the outer edge of the bed, and flows down by gravity. Inside of the bed, the liquid could appear in the form of thin films and tiny droplets because of high shear force, resulting in a large gas–liquid inter-facial area. The gas enters at the bottom, flows axially through the packing, reacts with the liquid, and leaves the bed from the gas outlet. Therefore, gas and liquid contact occurs in a cross-flow mode in the rotor.

#### 2.2.1. Preparation of PB in CF-RPB

**Figure 2:**
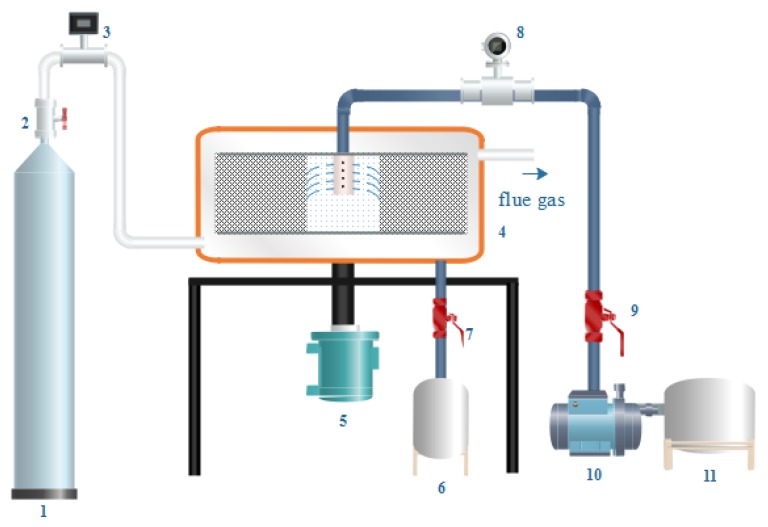
1—gas tank; 2, 7, 9—valve; 3—gas flow meter; 4—cross-flow rotating packed bed; 5—motor; 6, 11—liquid storage tank; 8—fluid flow meter; 10—pump.

The flow chart of the experiment is shown in Figure 2. The reaction occurred at room temperature (20 ± 2 °C). The volume of the NaAlO_2_ aqueous phase was 2 L. The optimal preparation conditions were obtained by investigating the gas–liquid ratio (0.5:1~4:1), the concentration of the NaAlO_2_ solution (0.05~0.6 mol/L), and the rotating speed of CF-RPB (200~1000 rpm).

The preparing process was as follows: CO_2_ gas passed through the flow meter and entered CF-RPB. Then, the NaAlO_2_ solution entered the CF-RPB through a peristaltic pump. The liquid flow rate was 50 L/h, and the gas–liquid ratio was controlled by changing the gas flow rate. After the gas flow stabilized, two phases were mixed and reacted by way of cross-flow until the NaAlO_2_ solution was run out. The products were flowed into tank 6 and recorded the final pH.

During this process, the nucleation and growth of PB particles were placed in two different reactors, that is, the nucleation of particles occured in CF-RPB, and the growth of particles was placed in a stirring tank reactor. Aging is an important process of particle growth, so when the reaction in CF-RPB stopped, the product was aged for 1 h in the stirring tank reactor at 70 °C, and then washed by ethanol and pure water repeatedly. The final product, PB powder, was obtained by drying at 100 °C for 6 h.

#### 2.2.2. Optimization of PB products

The preparation process of PB, including the nucleation and growth of particles, is accompanied by the aggregation, resulting in the reduction of internal channels. In order to make macromolecules easy to pass through the pores in the process of heavy oil catalysis and effective utilize the active sites, pore expansion modification on the preparation process of PB were carried out from the following two aspects:

Polyethylene glycol (PEG, [HO(–CH_2_CH_2_O-) n–H]) is one of the commonly used surfactants which has only two hydrophilic groups but no hydrophobic group. It can establish a strong hydrogen bond with PB to prevent the excessive growth of crystal particles. In this research, 5% PEG was mixed with NaAlO_2_ solution and reacted with CO_2_ to prepare PB in CF-RPB.

Secondly, the PB gel prepared by CF-RPB is improved by deionized water aging. Compared with the mother liquors aging, the impurities, such as Na^+^, can be removed and the residual sodium metaaluminate in the product can be prevented from hydrolysis in the process of initial filtration and washing. Therefore, the aging of purified water can effectively prevent the generation of impurities in the product.

### 2.3. Characterization

The crystal form of the prepared PB samples was determined by X-ray diffraction (XRD, DX-2700B, Dandong, China) at 45 kV using Cu Kα radiation with 2*θ* varying from 10° to 80°, a scan rate of 1°·min^–1^, and 2*θ* intervals of 0.02°. The specific surface area was obtained by the Brunauer–Emmett–Teller (BET) method. Pore size distribution and pore volume was calculated by the Barrett–Joyner–Halenda (BJH) method. The morphologies of PB were observed using scanning electron microscopy (SEM, JSM-7900F, Tokyo, Japan) and transmission electron microscopy (TEM, Hitachi H-600, Tokyo, Japan). The infrared spectra of PB were measured by a Fourier-transform infrared spectrometer (FT-IR, Perkin-Elmer, Waltham, Massachusetts, USA) to identify the nature of the bonding.

## 3. Results and Discussion

### 3.1. Preparation of PB in CF-RPB

#### 3.1.1. Effect of the Concentration of NaAlO_2_ Solution on PB Pore Properties

Table 2 shows the specific surface area, pore volume, and crystal form of the PB samples synthesized under different concentrations of NaAlO_2_ solution at a gas–liquid ratio of 1:1 and a CF-RPB rotating speed of 600 rpm. It can be observed, from Table 2, as the concentration of the NaAlO_2_ solution increased, the specific surface area and pore volume of the product increased first and then decreased. When the concentration of the NaAlO_2_ solution was 0.1 mol/L, the specific surface area and pore volume were the largest, with 455 m^2^/g and 0.589 cc/g, respectively. This may be due to the fact that when the concentration of NaAlO_2_ solution increases from 0.05 mol/L to 0.1 mol/L, the gas–liquid is fully reacted with the increase of the solution concentration, the crystallinity of the product becomes better, and the specific surface area and pore volume increase. When the concentration of the NaAlO_2_ solution continued to increase, the NaAlO_2_ in the reaction system was excessive, causing hydrolysis reaction to form Bayerite impurities, resulting in a decrease in specific surface area and pore volume of the product. When the concentration of the solution was 0.1 mol/L, the final pH of the PB formation was around 10.5, which is in accordance with the suitable final pH of the conventional carbonation method for preparing PB. In summary, the concentration of the NaAlO_2_ solution suitable for preparing PB is 0.1 mol/L.

**Figure 3:**
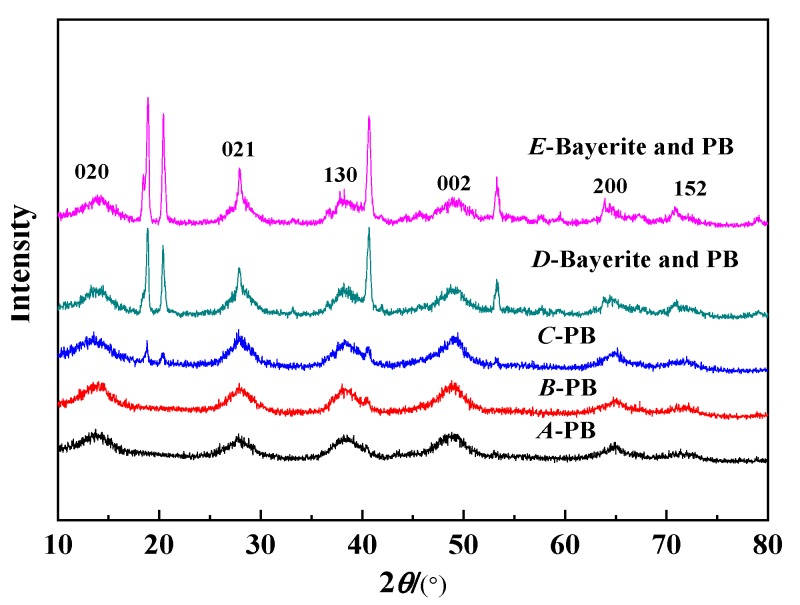
XRD results of PB with different concentrations of NaAlO_2_ solution (gas–liquid ratio. 1:1; rotating speed of CF-RPB, 600 rpm).

Figure 3 shows the XRD test results of the synthetic PB under different concentrations of NaAlO_2_ solution at a gas–liquid ratio of 1:1 and a CF-RPB rotating speed of 600 rpm. It can be seen from the figure that the products have obvious characteristic diffraction peaks at 2*θ* of about 13.9°, 28.3°, 38.5°, 49.2°, and 64.8°, which are characteristic diffraction peaks corresponding to PB [25]. When the concentration of the NaAlO_2_ solution increased from 0.05 mol/L to 0.1 mol/L, the XRD characteristic diffraction peak became sharp and the crystallinity became better. When the concentration of the NaAlO_2_ solution continued to increase, the product exhibited a distinct characteristic diffraction peak at 2*θ* of about 18° and 22°, which is a characteristic diffraction peak of Bayerite. The results in Figure 2 are consistent with the BET test results. It can be seen from Table 2 and Figure 2 that the PB with the best crystallinity and no impurity phase can be successfully obtained when the concentration of the NaAlO_2_ solution is 0.1 mol/L.

**Figure 4:**
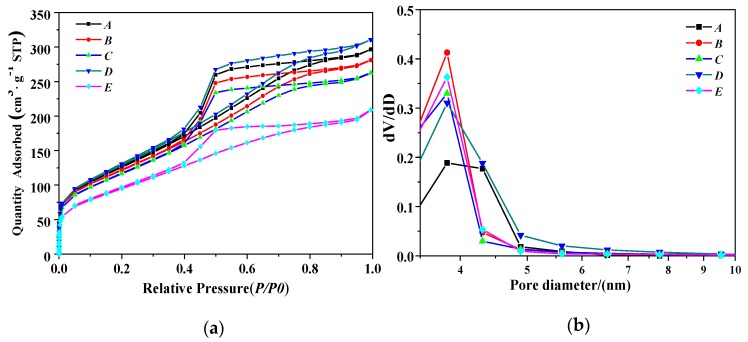
(**a**) N_2_ adsorption–desorption isotherm results of PB with different concentrations of NaAlO_2_ solution, (**b**) pore size distribution of PB with different concentrations of NaAlO_2_ solution (gas–liquid ratio, 1:1; rotating speed of CF-RPB, 600 rpm).

Figure 4a shows the N_2_ adsorption–desorption isotherm and the porosity types of PB samples synthesized under different concentrations of NaAlO_2_ solution at a gas–liquid ratio of 1:1 and a CF-RPB rotating speed of 600 rpm. As can be seen in Figure 4a, all isotherms are classical type IV [26], indicating that some pores have capillary condensation at higher pressures, which are essentially mesoporous materials. The hysteresis curves of these samples are between H2 and H3, indicating good pore connectivity [27,28]. Figure 4b shows the pore diameter distribution of PB prepared under this condition. It can be seen that the pore diameter distribution of PB is relatively narrow, ranging from 3 nm to 5 nm.

#### 3.1.2. Effect of the Gas–Liquid Ratio on PB Pore Properties

**Figure 5:**
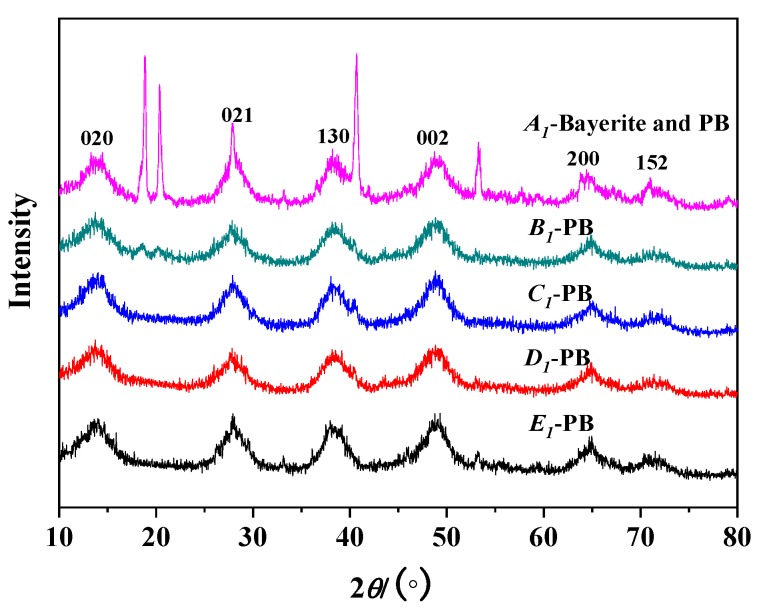
XRD results of PB with different gas–liquid ratios on pore properties (concentration of NaAlO_2_ solution, 0.1 mol/L; rotating speed of CF-RPB, 600 rpm).

The gas–liquid (G/L) ratio is the ratio of the flow rate of the CO_2_ gas to the NaAlO_2_ liquid flow, which is the ratio of the gas flow rate of the gas–liquid two phases in the instant contact. When the liquid flow rate is constant, the increase of gas volume will accelerate the reaction to the direction of product formation. The pore properties of PB produced by different gas–liquid ratios are shown in Table 3. When the gas–liquid ratio is 0.5:1, the specific surface area of the product is small and Bayerite impurities are formed.When the gas–liquid ratio is small, the CO_2_ is insufficient, which will easily lead to the hydrolysis of the NaAlO_2_ solution and the impurity of Bayerite is formed. As the gas–liquid ratio gradually increased, the specific surface area and pore volume of PB increased, which led to more and more CO_2_ gas that reacted with the NaAlO_2_ solution, a continuous increase in the purity of the product, and a reduction in the Bayerite impurity. As the gas–liquid ratio continues to increase to 4:1, the specific surface area and pore volume of the PB begin to decrease. When the gas–liquid ratio was 3:1, the product was pure PB with the highest specific surface area and the pore volume was 505 m^2^/g and 1.102 cc/g, respectively. This result corresponds to Figure 5: when the gas–liquid ratio was 0.5:1, there were strong miscellaneous peaks of Bayerite in the XRD pattern, which disappeared with the increase of the gas–liquid ratio.

**Figure 6:**
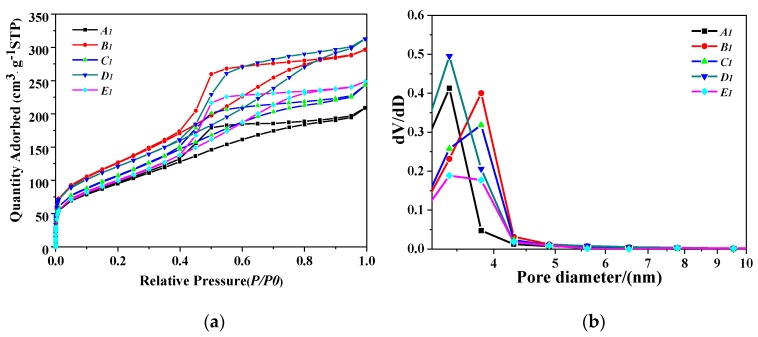
(**a**) N_2_ adsorption–desorption isotherm results of PB with different gas–liquid ratios, (**b**) pore size distribution of PB with different gas–liquid ratios (concentration of NaAlO_2_ solution, 0.1 mol/L; rotating speed of CF-RPB, 600 rpm).

Figure 6a shows the N_2_ adsorption–desorption isotherm and the porosity types of PB samples synthesized by different gas–liquid ratios. The hysteresis loops of PB are a composite of type H2 and H3, suggesting that they may have pore connectivity with channel-like or ink-bottle pores [29]. As the gas–liquid ratio increased, the pore volume also increased first and then decreased. When the gas–liquid ratio was 3:1, the higher closed point of the ring occurred above *P/P_0_* = 0.9, the adsorption amount was larger, and the pores were plate-like particles aggregated [30], consistent with the results shown in Figure 6b.

#### 3.1.3. Effect of the Rotating Speed of CF-RPB on PB Pore Properties

It can be seen from Table 4 that when the rotating speed of the CF-RPB was gradually increased, the specific surface area and the pore volume were gradually increased. When the rotating speed exceeded 600 rpm, the specific surface area and pore volume of the PB were gradually decreased. This phenomenon may be explained as follows: when the rotating speed is small, the gas–liquid contact area is small and the mixing is uneven, resulting in insufficient reaction of the CO_2_ gas with the NaAlO_2_ solution to generate a small amount of Bayerite impurities. When the rotating speed was gradually increased, the mass transfer between gas and liquid was strengthened, and the absorption rate of the CO_2_ gas was increased, so that the gas–liquid was fully reacted to produce a high-quality PB product. When the rotating speed increased to a certain extent, the gas–liquid mixture was fully mixed and it was no longer the main factor affecting mass transfer. It can be seen from Table 4 that the pore properties of the PB were the best when the rotating speed of CF-RPB reached 600 rpm.

**Figure 7:**
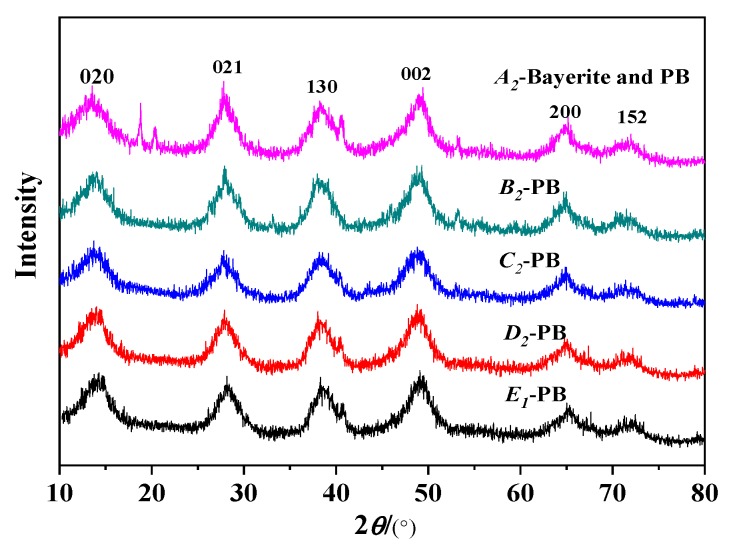
XRD results of PB with different rotating speeds of CF-RPB on pore properties (concentration of NaAlO_2_ solution, 0.1 mol/L; gas–liquid ratio, 3:1).

**Figure 8:**
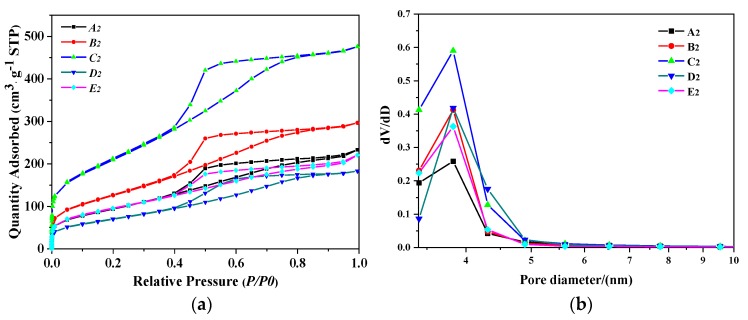
(**a**) N_2_ adsorption–desorption isotherm results of PB with different rotating speeds of CF-RPB, (**b**) pore size distribution of PB with different rotating speeds of CF-RPB (concentration of NaAlO_2_ solution, 0.1 mol/L; gas–liquid ratio, 3:1).

Figure 7 is an XRD pattern of PB produced under this condition, corresponding to the results of Table 4. Figure 8a shows the N_2_ adsorption–desorption isotherm results of PB with different rotating speeds of RPB, all isotherms are of classical type IV with a hysteresis loop suggesting the presence of mesopores. In Figure 8a, the nitrogen adsorption uptakes by PB samples synthesized under different rotating speeds are consistent with their pore volumes observed in Table 4. When the rotating speed was 600rpm, the adsorption amount and the pore volume were large. As the rotating speed increased, the hysteresis loop became H2 type, ending the bottom of the loop at the closing point of the horizontal platform, and the pore volume was reduced. It can be seen from Figure 8b that the pore diameter distribution of PB is relatively narrow, ranging from 3 nm to 5 nm.

The best preparation condition of PB can be obtained from the above experimental results: concentration of NaAlO_2_ solution, 0.1 mol/L; gas–liquid ratio, 3:1; rotating speed of CF-RPB, 600 rpm.

#### 3.1.4. Characterization and Analysis of PB Prepared under Suitable Conditions

**Figure 9:**
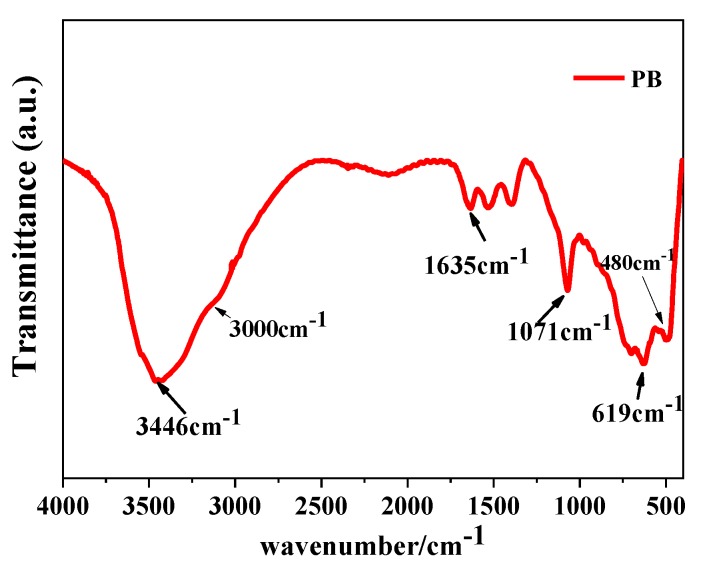
FT-IR results of PB (concentration of NaAlO_2_ solution, 0.1 mol/L; gas–liquid ratio, 3:1; rotating speed of CF-RPB, 600 rpm).

From the above experimental results, it is known that when the concentration of the NaAlO_2_ solution is 0.1 mol/L, the gas–liquid ratio is 3:1, and the rotating speed of CF-RPB is 600 rpm, mesoporous nano-PB with the largest specific surface area and pore volume is obtained. The PB obtained under this condition was analyzed by infrared spectroscopy, and Figure 9 is the FT-IR test result of the product under the stretching vibration peak of the C–O bond and Al–O bond [31].

**Figure 10:**
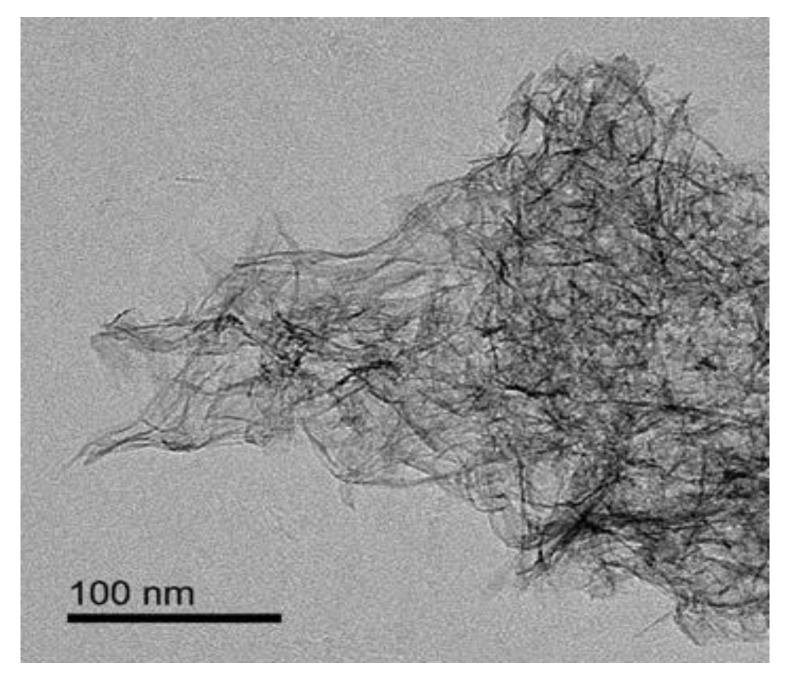
TEM morphology of the PB (concentration of NaAlO_2_ solution, 0.1.mol/L; gas–liquid ratio, 3:1; rotating speed of CF-RPB, 600 rpm).

Figure 10 shows the TEM morphology of the PB prepared under the following conditions: concentration of NaAlO_2_ solution, 0.1 mol/L; the gas–liquid ratio, 3:1; rotating speed of CF-RPB, 600 rpm. The morphology of PB showed that it presents a fluffy fibrous structure, which can provide a larger specific surface area. This shows that the products with the same morphology, uniform size, and high specific surface area can be prepared by CF-RPB.

### 3.2. Optimization of PB Products

**Figure 11:**
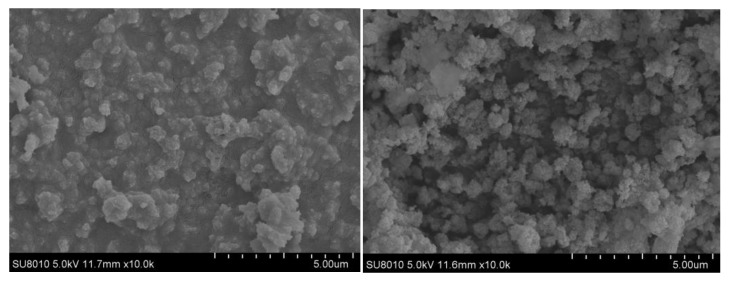
Comparison of SEM of PB before and after optimization.

Figure 11 shows the SEM of PB before and after optimization. It can be seen from the figure that the agglomeration of product particles and pore accumulation before optimization are relatively serious. The particle size of PB is uniform and dispersed after adding PEG surfactant and deionized water aging, and the pore channels are rich. Through BET analysis of the optimized PB products in Table 5, it can be seen that the pore volume of the optimized PB increased from 1.102 cc/g to 2.125 cc/g, and the specific surface area did not decrease significantly. This result also corresponds to SEM, which fully proves the feasibility of the preparation of nano-PB with a large pore volume and high specific surface area by high-gravity continuous carbonation.

### 3.3. Comparison and Application

#### 3.3.1. The Comparison of Different Methods for Preparing PB

Table 6 is a comparison of the preparation of PB by different methods. According to the table, the specific surface area and pore volume of the PB prepared in the traditional batch reactor are small with a long handling time. Compared with batch carbonation in RPB, the specific surface area and pore volume of PB produced by continuous carbonation in RPB greatly improved. This method not only maintains a higher specific surface area and pore volume, but also improves product purity compared with three other methods. At the same time, the continuous carbonation in RPB has a short processing time and is suitable for industrial applications. In this process, as long as a fixed gas–liquid ratio is maintained, the volume of gas and liquid injected by amplification will not significantly increase the processing time.

#### 3.3.2. The Difference of Batch and Continuous Carbonation Processes in RPB

In this paper, the PB is produced by a continuous process. The entire reaction process can be regarded as a process of equal pH feeding. This indicates that the gas–liquid reaction remains a stable pH during the reaction process, which effectively ensures the purity of the product and is also one of the advantages of continuous production. Table 7 is the final pH of a PB preparation process optimized for NaAlO_2_ concentration, gas–liquid ratio, and CF-RPB rotation speed. It can be assumed that the final pH of PB prepared under the optimum preparation conditions is around 10.5, which is in line with the suitable final pH of preparing the PB by the traditional carbonation method.

## 4. Conclusions

In this paper, a cross-flow rotating packed bed is used as a carrier to provide a high-gravity environment. The NaAlO_2_–CO_2_ carbonation method was combined with the continuous production process. Under the condition of the gas–liquid ratio being 3:1, the rotating speed of CF-RPB being 600 rpm, and the concentration of the NaAlO_2_ solution being 0.1 mol/L, fibrous pseudo-boehmite, with a specific surface area of 505 m^2^/g and pore volume of 1.102 cc/g, was successfully obtained. After pore expansion and modification, macroporous pseudo-boehmite with a specific surface area of 495 m^2^/g and pore volume of 2.125 cc/g was obtained. The advantages of RPB and continuous carbonation technology are combined. Compared with the traditional stirred tank reactor, the high-gravity continuous carbonation production of PB has the advantages of high production efficiency, stable product quality, small equipment volume, simple operation and maintenance, and low energy consumption, which is expected to be utilized for industrial application.

## Figures and Tables

**Table 1:** The equipment parameters of the cross-flow rotating packed bed (CF-RPB).

Character	Parameter
Packing type	Stainless steel wire meshes of 0.3 mm
Outer diameter of packing	190 mm
Inner diameter of packing	56 mm
Axial height of packing	100 mm

**Table 2:** The pore properties of pseudo-boehmite (PB) under different concentrations of NaAlO_2_ solution (gas–liquid ratio, 1:1; rotating speed of CF-RPB, 600 rpm).

Number	NaAlO_2_ Concentration	Final pH	Crystal Form	S (m^2^/g)	V (cc/g)
*A*	0.05	10.5	PB	432	0.412
*B*	0.1	10.5	PB	455	0.589
*C*	0.2	11	PB + Bayerite	381	0.451
*D*	0.4	11	PB + Bayerite	359	0.323
*E*	0.6	11.5	PB + Bayerite	295	0.197

**Table 3:** The pore properties of PB under different gas–liquid ratios (concentration of NaAlO_2_,0.1 mol/L; rotating speed of CF-RPB, 600 rpm).

Number	G/L	Final pH	Crystal Form	S (m^2^/g)	V (cc/g)
*A_1_*	0.5:1	11	PB + Bayerite	343	0.435
*B_1_*	1:1	10.5	PB	455	0.789
*C_1_*	2:1	10.5	PB	469	0.825
*D_1_*	3:1	10.5	PB	505	1.102
*E_1_*	4:1	9.5	PB	402	0.578

**Table 4:** Effect of the rotating speed of CF-RPB on pore properties of PB (concentration of NaAlO_2_ solution, 0.1 mol/L; gas–liquid ratio, 3:1).

Number	CF-RPB Rotate Speed (rpm)	Final pH	Crystal Form	S (m^2^/g)	V (cc/g)
*A_2_*	200	11	PB + Bayerite	335	0.282
*B_2_*	400	10.5	PB	432	0.458
*C_2_*	600	10.5	PB	505	1.012
*D_2_*	800	10.5	PB	420	0.645
*E_2_*	1000	10.5	PB	380	0.593

**Table 5:** Comparison of pore properties of PB before and after optimization.

	S (m^2^/g)	V (cc/g)
Before optimization	505	1.102
After optimization	495	2.125

**Table 6:** Comparison of different methods for preparing PB.

Equipment.	S	V	Handling Capacity	Handling Time	Final pH
	(m^2^/g)	(cc/g)	(L)	(min)	
RPB batch carbonation	437	-	3	7.4	10.5
RPB continuous carbonation	495	2.125	2	0.8	10.5
Stirring tank reactor	316	0.35	2	12	10.5
Membrane-dispersion Microstructured reactor	548	2.22	3.5	7	10.5

**Table 7:** The final pH of PB prepared under suitable operating conditions.

Number	NaAlO_2_ Concentration	G/L	CF-RPB Rotate Speed (rpm)	Final pH
*B*	0.1	1:1	600	10.5
*C*_2_	0.1	3:1	600	10.5
